# First report of *Enterococcus thailandicus* isolated from routine vancomycin-resistant enterococci (VRE) screening samples

**DOI:** 10.1016/j.nmni.2026.101712

**Published:** 2026-01-25

**Authors:** Dan-Alexandru Toc, Alexandru Botan, Ioana Colosi, Carmen Costache

**Affiliations:** a Department of Microbiology, Iuliu Hatieganu University of Medicine and Pharmacy, 400012, Cluj-Napoca, Romania; b Cluj County Emergency Hospital, 400000, Cluj-Napoca, Romania; c Faculty of Medicine, Iuliu Hatieganu University of Medicine and Pharmacy, 400012, Cluj-Napoca, Romania; d Division of Medical Informatics and Biostatistics, Department of Medical Education, Iuliu Hatieganu University of Medicine and Pharmacy, 400012, Cluj-Napoca, Romania

**Keywords:** *Enterococcus thailandicus*, Vancomycin-resistant enterococci, VRE screening, Antimicrobial susceptibility, Rare enterococci

Dear Editor,

*Enterococcus thailandicus* remains one of the least characterized members of the genus *Enterococcus* in clinical microbiology. Originally described under two different names (*Enterococcus sanguinicola* from human blood cultures and *E. thailandicus* from fermented food) this species has since been unified taxonomically, yet its epidemiology, antimicrobial susceptibility profile, and diagnostic relevance remain poorly defined [1,2]. In this context, we wish to highlight the recovery and characterization of five *E. thailandicus* isolates identified during routine vancomycin-resistant enterococci (VRE) screening in an intensive care unit, emphasizing important diagnostic and epidemiological implications.

All five isolates were recovered from rectal swabs plated on chromogenic VRE agar (bioMérieux, Marcy-l’Étoile, France) as part of routine surveillance protocols. Despite growth on selective VRE media, subsequent identification by MALDI Biotyper® Sirius system (Bruker Daltonics, Bremen, Germany) and antimicrobial susceptibility testing, using Sensititre™ EUENCF plates (Thermo Fisher Scientific, Waltham, MA, USA), demonstrated preserved susceptibility to glycopeptides, with uniformly low minimum inhibitory concentrations (MICs) for both vancomycin and teicoplanin. This finding underscores a critical limitation of chromogenic VRE screening media, namely reduced specificity when applied to uncommon or poorly characterized enterococcal species [3]. The recovery of vancomycin-susceptible *E. thailandicus* from VRE plates represents a clear example of false-positive screening that may lead to unnecessary infection control measures or misinterpretation of local VRE epidemiology.

Phenotypically, the isolates displayed a largely conserved antimicrobial susceptibility profile (Table 1). All strains remained susceptible to β-lactam agents as well as to linezolid. These findings are concordant with previously reported human isolates, which consistently demonstrated preserved susceptibility to these agents [4,5]. In contrast, fluoroquinolone susceptibility was heterogeneous, with one isolate exhibiting high-level resistance to ciprofloxacin and levofloxacin, suggesting strain-dependent acquisition of resistance mechanisms affecting DNA gyrase or topoisomerase IV. Additionally, all isolates showed high MICs to nitrofurantoin, a finding of particular clinical relevance given the frequent empirical use of this agent in urinary tract infections.

**Table 1:** Antimicrobial susceptibility testing results of the 5 *Enterococcus thailandicus* strains (interpretation perfomed using EUCAST guideline).

				*E. thailandicus 1*	*E. thailandicus 2*	*E. thailandicus 3*	*E. thailandicus 4*	*E. thailandicus 5*
1.	Ampicilin	CMI		0,5	0,5	0,5	0,5	1
		Interpretation	i.v.	S	S	S	S	S
2.	Amoxacilin	CMI		≤0,25	≤0,25	≤0,25	≤0,25	0,5
		Interpretation	i.v.	S	S	S	S	S
			oral (1)	S	S	S	S	S
3.	Amoxacilin/Clavulanic acid	CMI		≤0,25	≤0,25	≤0,25	≤0,25	0,5
		Interpretation	i.v.	(2)	(2)	(2)	(2)	(2)
			oral (1)	(2)	(2)	(2)	(2)	(2)
4.	Imipenem	CMI		≤0,5	≤0,5	1	≤0,5	2
		Interpretation	(3)	S	S	S	S	S
5.	Vancomicin	CMI		0,5	0,5	0,5	0,5	1
		Interpretation		S	S	S	S	S
6.	Teicoplanin	CMI		≤0,5	≤0,5	≤0,5	≤0,5	≤0,5
		Interpretation		S	S	S	S	S
7.	Linezolid	CMI		4	4	2	4	2
		Interpretation		S	S	S	S	S
8.	Tigecyclin	CMI		0,25	0,25	≤0,06	0,12	0,12
		Interpretation		S	S	S	S	S
9.	Ciprofloxacin	CMI		1	1	1	1	16
		Interpretation	(1)	S	S	S	S	R
10.	Levofloxacin	CMI		2	2	2	8	16
		Interpretation	(1)	S	S	S	R	R
11.	Norfloxacin	CMI		>16	8	>16	≤4	>16
		Interpretation		n/a	n/a	n/a	n/a	n/a
12.	Streptomycin	CMI		≤512	≤512	≤512	≤512	≤512
		Interpretation		SYN-S	SYN-S	SYN-S	SYN-S	SYN-S
13.	Gentamicin	CMI		≤32	≤32	≤32	≤32	≤32
		Interpretation		SYN-S	SYN-S	SYN-S	SYN-S	SYN-S
14.	Quinupristin/Dalfopristin	CMI		2	2	2	2	8
		Interpretation	(4)	R	R	R	R	R
15.	Nitrofurantoin	CMI		>64	>64	>64	>64	>64
		Interpretation	(1)(5)	R	R	R	R	R
16.	Trimethoprim	CMI		0,06	0,06	0,06	0,06	4
		Interpretation	(1)	(6)	(6)	(6)	(6)	(6)

n/a = not available.

SYN-S = synergy with penicillins or glycopeptides can be expected if the isolate is susceptible to the penicillins or glycopeptides.

(1) = uncomplicated UTI only.

(2) = susceptibility can be inferred from ampicilin.

(3) = based on PK/PD.

(4) = based on *E. faecium* breakpoint.

(5) = based on *E. faecalis* breakpoint.

(6) = The activity of trimethoprim is uncertain against enterococci, and it is not possible to predict clinical outcome. Isolates with MICs >1 mg/L most likely have resistance mechanisms against the antibiotic.

The recovery of *E. thailandicus* from VRE screening plates raises broader questions regarding the interpretation of surveillance cultures. While selective media are indispensable tools for infection control, their performance characteristics are typically validated for *E. faecium* and *E. faecalis*, with limited data on rare enterococcal species [3]. As diagnostic laboratories increasingly encounter non-*faecalis*, non-*faecium* enterococci, confirmatory identification and susceptibility testing become essential to avoid misclassification and overestimation of VRE carriage.

Although limited by the small number of isolates and the absence of whole-genome sequencing, these observations add meaningful data to the sparse literature on *E. thailandicus*. The consistent glycopeptide susceptibility, combined with occasional acquisition of mobile resistance genes, suggests a species that is currently of low clinical virulence but of potential epidemiological importance. We believe that continued reporting of such findings is essential to refine diagnostic algorithms, improve interpretation of screening results, and better understand the role of rare enterococci in antimicrobial resistance ecology.

## CRediT authorship contribution statement

**Dan-Alexandru Toc:** Conceptualization, Data curation, Investigation, Methodology, Project administration, Validation, Writing – review & editing, Writing – original draft. **Alexandru Botan:** Data curation, Formal analysis, Methodology, Software, Validation, Visualization. **Ioana Colosi:** Funding acquisition, Formal analysis, Supervision, Validation, Writing – review & editing, Writing – original draft. **Carmen Costache:** Writing – review & editing, Writing – original draft, Validation, Supervision, Project administration, Funding acquisition, Formal analysis.

## Declaration of competing interest

The authors declare that they have no known competing financial interests or personal relationships that could have appeared to influence the work reported in this paper.

## References

[1] Tanasupawat S., Sukontasing S., Lee J.S. (Jul. 2008). Enterococcus thailandicus sp. nov., isolated from fermented sausage ('mum’) in Thailand. Int J Syst Evol Microbiol.

[2] Shewmaker P.L. (2011). Reevaluation of the taxonomic status of recently described species of enterococcus: evidence that E. thailandicus is a senior subjective synonym of ‘E. sanguinicola’ and confirmation of E. caccae as a species distinct from E. silesiacus. J Clin Microbiol.

[3] Ledeboer N.A. (May 2007). Evaluation of a novel chromogenic agar medium for isolation and differentiation of vancomycin-resistant Enterococcus faecium and Enterococcus faecalis isolates. J Clin Microbiol.

[4] Tsuda Y. (Dec. 2025). Case report of *Enterococcus thailandicus* bacteremia, with genomic characterization of its clinical isolate. ASM Case Rep.

[5] Mbouche P. (Jun. 01, 2023).

